# Electrospun Nanofibres Containing Antimicrobial Plant Extracts

**DOI:** 10.3390/nano7020042

**Published:** 2017-02-15

**Authors:** Wanwei Zhang, Sara Ronca, Elisa Mele

**Affiliations:** Department of Materials, Loughborough University, Loughborough LE11 3TU, UK; w.zhang4@lboro.ac.uk (W.Z.); s.ronca@lboro.ac.uk (S.R.)

**Keywords:** plant extracts, electrospinning, wound healing, tissue engineering, food industry

## Abstract

Over the last 10 years great research interest has been directed toward nanofibrous architectures produced by electrospinning bioactive plant extracts. The resulting structures possess antimicrobial, anti-inflammatory, and anti-oxidant activity, which are attractive for biomedical applications and food industry. This review describes the diverse approaches that have been developed to produce electrospun nanofibres that are able to deliver naturally-derived chemical compounds in a controlled way and to prevent their degradation. The efficacy of those composite nanofibres as wound dressings, scaffolds for tissue engineering, and active food packaging systems will be discussed.

## 1. Introduction

The electrospinning technology, short for electrostatic spinning, has attracted great interest in recent decades, thanks to its capability of easily and effectively processing a huge range of polymeric materials in form of nanofibres [1]. Compared with other processes for nanofiber fabrication, such as self-assembly and phase separation [2], electrospinning has the advantages of versatility and flexibility, in terms of material selection and control over fibres’ morphology and diameter [3]. Moreover, it offers the possibility to form various fibre networks, including nonwoven, aligned, or patterned fibre meshes, randomly distributed three dimensional (3D) structures, sub-micron spring and convoluted fibres [3]. These characteristics, together with the extremely high surface to volume ratio, make electrospun fibres the nanomaterials of election in applications such as sensors, energy, biomedicine, and filtration [4,5].

In recent years, the health concerns associated with the side effects of synthetic compounds used in cosmetics, medicine, and food industry and the emergence of antibiotic resistance of pathogens have driven electrospinning research towards the development of fibres encapsulating plant extracts [6]. In fact, plants are sources of many chemical compounds that exhibit antimicrobial activity [7,8] and have the potential to treat antimicrobial-resistant infections [9,10]. The work of Cowan discusses in detail the diverse classes of antimicrobial phytochemicals synthesised by plants [11], such as phenolics, terpenoids, and alkaloids. The mechanisms of action of those compounds against pathogens are various and include disruption of the cell membrane, complex with cell wall, substrate deprivation, and enzyme inhibition.

**Figure 1:**
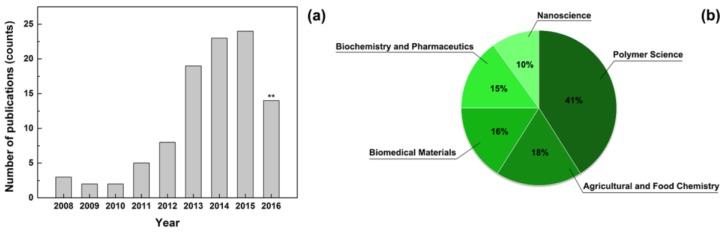
(**a**) Number of papers published per year on electrospun nanofibres containing plant extracts. ** This count considers the first ten months of 2016; (**b**) Analysis of the results by subject area. Scopus database was used to determine the total number of publications, searching for “electrospinning” plus “plant extract” or “essential oil”.

The last 10 years have seen an increased number of publications on fibres produced by electrospinning a variety of plant extracts (Figure 1). The current total number of articles on this topic (around 100) is low if compared with the number of papers published every year on electrospinning, but it is expected to rise further in the coming years. This review will discuss the recent achievements on electrospinning of antibacterial plant extracts, focusing on the application of the resulting composite fibres in regenerative medicine and food industry.

## 2. The Electrospinning Process

**Figure 2:**
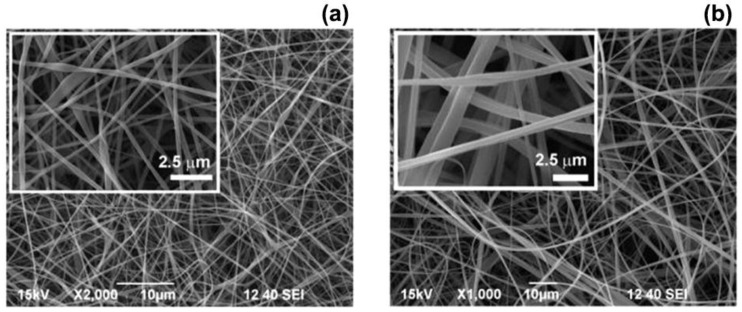
Scanning electron microscope images of electrospun fibres of (**a**) fluoroacrylic copolymer and (**b**) cellulose acetate. Reproduced with permission from [13]. Copyright American Chemical Society, 2014.

The electrospinning process is an electro-hydrodynamic phenomenon based on the extrusion and stretching of thin filaments of polymer solutions or melts by the application of an electric force [12]. The main components of an electrospinning apparatus are a supply system, a collector, and a high voltage power generator. The supply system is typically a metallic needle connected to a syringe filled with a solvent-based polymer solution (solution electrospinning) or with a molten polymer (melt electrospinning). In both cases, when a voltage in the range of 5–60 kV is applied between the needle and the grounded metallic collector, electrical charges are generated on the surface of the polymer droplet at the end of the needle. The droplet consequently deforms and acquires a cone-like shape (known as a Taylor cone). If the electric force overcomes the liquid surface tension, a polymeric filament is extruded at the tip of the Taylor cone and it is attracted towards the collector. In this phase, the filament further elongates and the evaporation of the solvent takes place, resulting in a network of non-woven fibres on the surface of the collector [13] (Figure 2).

The electrospinning of a wide range of materials—including synthetic and natural polymers, proteins, composites, and nanocomposite systems—has been demonstrated so far [6], proving that the size and the morphology of the electrospun fibres can be controlled by acting on the material viscosity and conductivity, and on the operational parameters (voltage, needle-collector distance, environmental humidity, and temperature). Additionally, an aspect that is relevant for the formation of fibres during electrospinning is the entanglement of the polymer chains, which is associated with polymer molecular weight and solution concentration [14,15]. When the chain entanglement is insufficient to stabilise the liquid jet, beads, or not-uniform fibres are formed. On the contrary, an appropriate level of chain entanglement promotes the generation of a jet that is stable during the solvent evaporation and leads to fibrous structures [16,17]. For instance, it has been demonstrated that, for solutions of poly(methyl methacrylate) (PMMA) in chloroform and dimethyl formamide (DMF), the elastic stretching of the polymer network and the entanglement loss determine fragmentation of the electrospun jet, resulting in short nanofibres [18].

The electrostatic force applied during the electrospinning process is not only responsible for fibre formation but it has an effect also on the orientation of the polymer chains along the main fibre axis [19,20]. A recent study on polyethylene (PE) nanofibres has demonstrated that the level of molecular orientation was dependent on the intensity of the electric field used [21]. When PE was electrospun with a voltage of 45 kV, high crystallinity was achieved, as demonstrated by micro-Raman spectroscopy. Moreover, the thermal conductivity of the fibres increases by increasing the voltage: thermal conductivity of 9.3 W·m^−1^·K^−1^ was obtained at 45 kW that was over 10 times higher than the thermal conductivity of PE fibres electrospun at 9 kV (0.8 W·m^−1^·K^−1^). This was clearly linked with a higher degree of orientation of the polymer chains that allowed thermal conduction through lattice movements in an electrically insulating polymer. Previous studies have shown that highly oriented nanofibers of ultra-high molecular weight polyethylene (UHMWPE) can reach conductivities of 104 W·m^−1^·K^−1^ [22] while values as high as 46 W·m^−1^·K^−1^ have been obtained for tapes of oriented UHMWPE [23]. In the specific case of UHMWPE, the high amount of chain entanglements leads to highly viscous solutions [24] that can be difficult to electrospin. Strategies based on the use of very diluited solutions [25,26] and ‘disentangled’ UHMWPE [27,28] can be adopted to overcome this limitation.

By controlling the materials’ properties and processing parameters, the electrospinning technique offers the possibility to produce fibres that possess the desired physicochemical properties, are characterised by a multiscale hierarchical structure (fibre’s surface decorated with pores, knots, and papillae) and are able to encapsulate and release functional chemical compounds [29]. They can find application in diverse sectors, including tissue engineering, pharmaceutics, energy, filtration, and food industry. In the following sections, the development of composite fibres by electrospinning natural antimicrobial agents will be discussed with particular focus on advanced wound dressings, scaffolds for regenerative medicine, and smart food packaging systems.

## 3. Electrospun Antibacterial Dressings

The electrospinning of naturally derived bioactive agents has been proposed for the development of advanced dressings able to promote rapid and efficient wound repair [30,31,32,33,34,35,36,37,38,39,40,41,42,43,44,45,46,47,48,49,50,51]. In fact, electrospun scaffolds possess several advantages over traditional dressings for the treatment of chronic and acute wounds [52,53]: high absorption of exudates from the wound site, efficient exchange of gases and nutrients to promote cells proliferation, physical protection of the injured tissue, and the possibility to release functional molecules. This section will discuss recent achievements in the field of nanofibrous dressings loaded with crude plant extracts, essential oils, and plant-derived chemical components.

### 3.1. Crude Plant Extracts

Crude extracts are easily obtained by organic solvent extraction from fresh plants or from milled dried plants. Several crude plant extracts have been successfully encapsulated into electrospun fibres, such as *Centella asiatica* [39], baicalein [37], green tea [36], *Garcinia mangostana* [38,42], *Tecomella undulata* [32], aloe vera [44,54], *Grewia mollis* [55], chamomile [35], grape seed [33], *Calendula officinalis* [56], *Indigofera aspalathoides*, *Azadirachta indica*, *Memecylon edule* and *Myristica andamanica* [43].

Yao and co-workers have produced gelatin nanofibres loaded with *Centella asiatica* (CA), a medicinal herb from Asia that is composed of triterpenoids (such as Asiatic acid, madecassic acid and asiaticoside) and has wound healing activity [39]. The authors demonstrated that the CA extract was effective against *Staphylococcus aureus*, *Escherichia coli*, and *Pseudomonas aeruginosa* (bacteria that are commonly implicated in wound infections) with a minimum inhibitory concentration (MIC) of 6.25 mg/mL for *S. aureus* and 25 mg/mL for *E. coli* and *P. aeruginosa*. They observed also that the electrospun gelatin CA membranes (EGC) with 31.2 mg/mL of CA extract promoted the proliferation of fibroblasts (up to 93% of cell viability). In animal tests, wounds treated with EGC membranes recovered of 83% in seven days and were characterised by an increased formation of blood capillaries. Overall, the results collected proved that CA has a positive effect in accelerating wound healing, by acting on inflammatory and proliferative phases and protecting the injured site against bacteria colonisation. Another herbal extract that has been used for wound care applications is baicalein, a Chinese herb with antibacterial, anti-inflammatory, and antioxidant properties [37]. Baicalein (BAI) was incorporated into silk fibroin (SFP) and polyvinylpyrrolidone (PVP) fibres and its antibacterial activity was evaluated in vitro against *S. aureus*. Differently from SFP/PVP fibres, SFP/PVP/BAI mats exhibited a reduction of bacteria viability in the range of 88%–99%. More importantly, in vivo studies on wounds infected with *S. aureus* showed acceleration of the wound closure with a complete recovery after 21 days. The analysis of the pus collected from the wound seven days after the bacterial inoculation had a reduced load of microorganisms (2.8 × 10^8^ CFU/g) for the animals treated with SFP/PVP/BAI membranes, whereas the load was of 2.3 × 10^9^ CFU/g in the case of SFP/PVP fibres. The comparison with a commercial dressing (Tegaderm) proved that the composite electrospun mats had superior performance in terms of skin re-epithelialisation, collagen synthesis, and wound closure.

Sadri and collaborators have instead analysed the effect of green tea extract electrospun in a matrix of chitosan and polyethylene oxide (PEO) [36]. In vitro antibacterial tests showed the efficacy of the composite chitosan-PEO/green tea fibres against *E. coli* and *S. aureus*, with inhibition zones on agar plates of 4 and 6 mm in diameter, respectively. On the contrary, chitosan-PEO nanofibres did not exhibit any activity. An animal model of infected wound was used to test the composite nanofibrous dressings in vivo, proving that the animals completely recovered after 16 days, differently from animals treated with chitosan-PEO fibres. Chitosan has been proposed also as matrix for the electrospinning of extracts of *Garcinia mangostana* (GM), a tropical fruit found in Southeast Asia [42]. GM was loaded into electrospun mats of chitosan-ethylenediaminetetraacetic acid/polyvinyl alcohol (CS-EDTA/PVA) and its release was studied in vitro. Results showed a burst release of GM from the fibres (80% within 60 min) due to the swelling of chitosan in water. The fibres produced were able to inhibit the growth of *S. aureus* and *E. coli*, with a concentration dependent efficacy: CS-EDTA/PVA mats loaded with 1, 2, and 3 wt % of GM extracts exhibited a MIC of 2.0, 1.0, and 0.5 mg/mL, respectively, against both *S. aureus* and *E. coli*. The wound healing activity of the electrospun mats was demonstrated on an animal model of open wound, obtaining that fibres with 3 wt % of GM extracts induced a full recovery within 11 days with skin reepithelialisation and replacement of granulation tissue by hair follicles.

**Figure 3:**
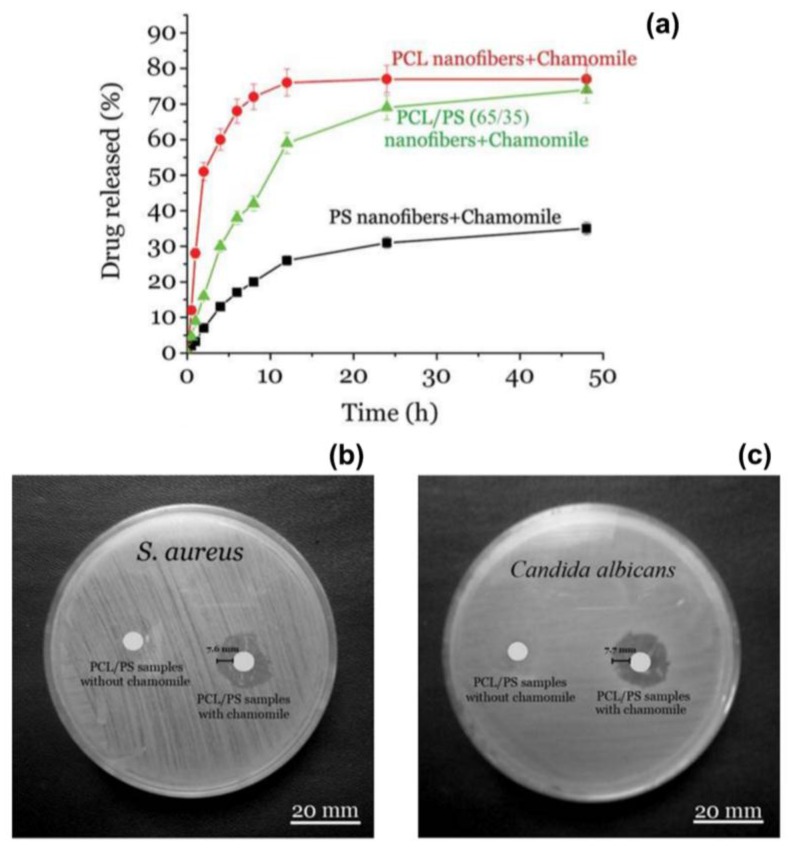
(**a**) Release profile of chamomile from PCL (red circles), PCL/ polystyrene (PS) (green triangles) and PS (black squares) electrospun nanofibres over a time period of 48 h. PS samples showed a lower release if compared with PCL and PCL/PS samples. Antibacterial and antifungal properties of the composite PCL/PS fibre loaded with chamomile against (**b**) *S. aureus* and (**c**) *C. albicans*. Adapted with permission from [35].

Suganya et al. have proposed the use of nanofibres of polycaprolactone (PCL) and PVP embedding crude bark extracts of *Tecomella undulata*, a medicinal plant from Thar Desert regions of northwest and western India, for the treatment of skin infections [32]. The extract was rapidly released in vitro from the composite mats in the first few hours, followed by a slow release over a prolonged period of time (24 h). The potent antibacterial activity of the bark extracts released from the electrospun mats was evaluated against *P. aeruginosa*, *S. aureus*, and *E. coli*, achieving zones of inhibition of 30, 24, and 28 mm in diameter, respectively. Finally, Motealleh and co-workers reported on PCL/polystyrene (PS) nanofibrous mats containing chamomile, a medicinal plant of the Asteraceae family (Figure 3a) [35]. The PCL/PS/chamomile fibres exhibited an inhibition effect against bacteria (*S. aureus*) and fungi (*Candida albicans*), as shown in Figure 3b,c. Moreover, they were able to heal wounds in an animal model leading to fibroblast proliferation, re-epithelization, and increased formation of granulation tissue.

### 3.2. Essential Oils

Essential oils (EOs) are typically extracted from aromatic plants and, similar to crude plant extracts, are mixtures of different chemical compounds, such as linalool, pinene, eugenol, and cymene [57]. As discussed by Burt [57] and confirmed by other studies [58,59], the mechanism of action of EOs against microorganisms is determined by their hydrophobic nature. EOs can be partitioned into the lipid bilayer of the bacteria cell membrane, with a consequent disruption of its structure. The membrane is then made permeable to ions and other cellular content, and this eventually leads to the collapse of the proton pump and to cell death.

The bioactivity of EOs has been exploited in combination with electrospinning in order to produce fibrous scaffolds that possess antibacterial properties and, in some cases, controlled thermo-mechanical properties. EOs that have been electrospun include Candeia [48], cinnamon [46,60], lemongrass and peppermint [46], tea tree [49], thyme [47], and lavender [50]. Rieger et al. demonstrated the production of electrospun chitosan/PEO nanofibres containing cinnamon EO [51]. Fibres with a diameter in the range of 38–55 nm were produced by electrospinning chitosan and PEO (1:1 ratio) from aqueous solutions containing 5 *w*/*v* % of acetic acid and different concentrations of EO (0.5 and 5.0 *v*/*v* %). After production, the fibres were cross-linked using glutaraldehyde, in order to increase their chemical stability. The chitosan/PEO nanofibres were tested against *P. aeruginosa* achieving a rate of bacteria inactivation of 50% and 76% for mats without and with 5% of cinnamon EO, respectively. Moreover, the composite nanofibres were active against *E. coli* after 30 min of contact with the bacteria and a viability loss higher than 99% was measured after 180 min. The efficacy of cinnamon EO against *E. coli* has been demonstrated also in another study, where cellulose acetate has been used as polymer matrix [46]. In the same work, fibres containing peppermint and lemon grass EOs have been analysed as well, concluding that the scaffolds produced were able to inhibit the proliferation of *E. coli*, while being not cytotoxic for fibroblasts and keratinocytes.

**Figure 4:**
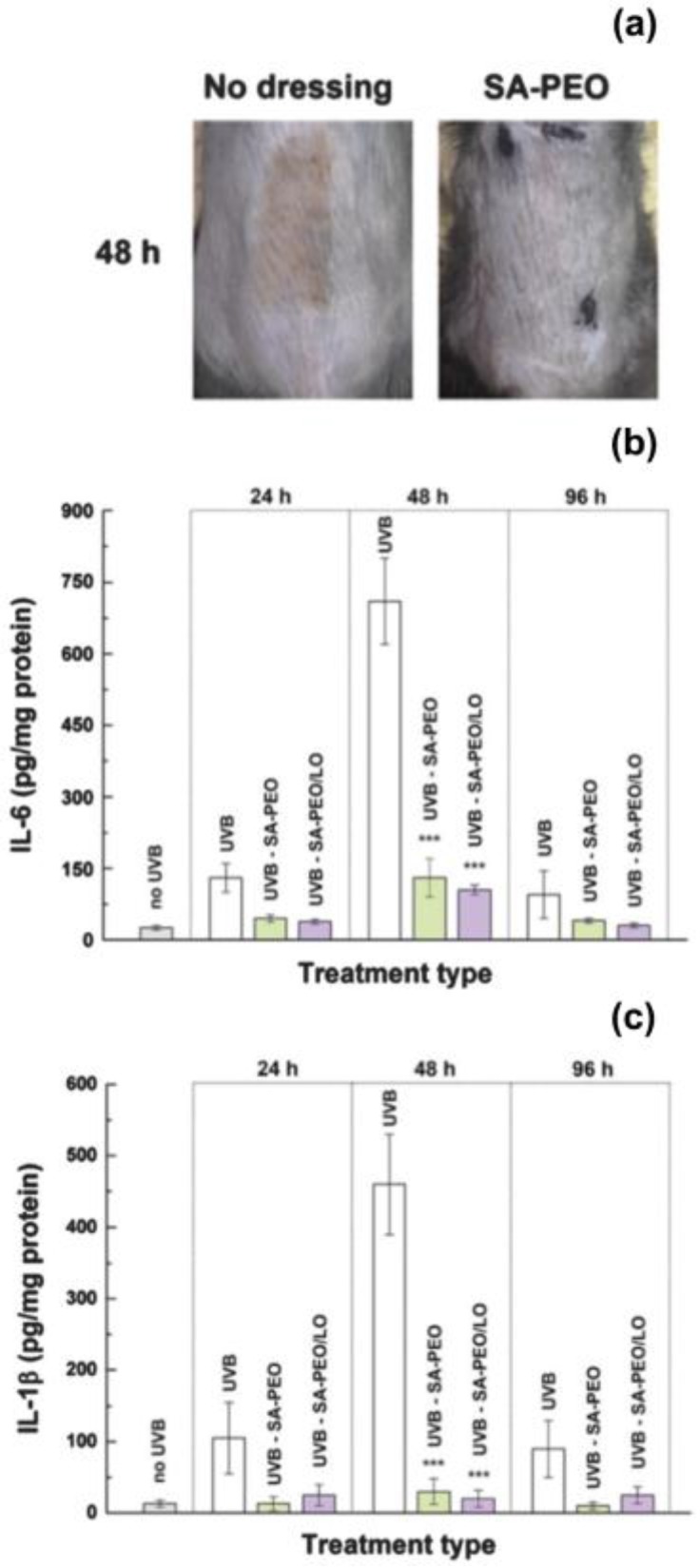
(**a**) Photographs of the skin of a mice 48 h after irradiation with ultraviolet light: untreated (no dressing) and treated with alginate (SA)-poly(ethylene oxide) (PEO) fibres (SA-PEO). An evident burn mark (red area) was visible for the animals without treatment, differently from mice treated with the electrospun dressings (no trace of erythema). Time course of the expression of (**b**) Interleukin-6 (IL-6) and (**c**) Interleukin-1β (IL-1β) for animals without treatment (UVB) and for animals treated with SA-PEO fibres (UVB-SA-PEO) and with SA-PEO fibre containing lavender essential oil (LO) (UVB-SA-PEO/LO). Adapted with permission from [50].

Balasubramanian et al. have directed their attention towards *Syzygium aromaticum* (clove) oil, which is mainly composed of eugenol and caryophyllene and therefore it is able to act on the cell wall biosynthesis and on the primary metabolism of bacteria [61]. The oil has been encapsulated into electrospun mats of polyacrylonitrile (PAN) having an average diameter of about 144 nm. The investigations performed on Gram positive (*S. aureus* and *Bacillus subtilis*) and Gram negative bacteria (*Klebsiella pneumonia* and *E. coli*) showed zones of inhibition with a diameter of 2.5–2.8 cm and 1.8–2.0, respectively. Hence, the fibres had limited efficacy against Gram negative bacterial strains, probably because these possess an extra outer membrane with a bilayer phospholipid structure that protects the cytoplasmic membrane of the cells. The authors have also used PAN nanofibres in combination with lavender essential oil, measuring a zone of inhibition of 14–15 mm in diameter for *S. aureus* and *K. pneumonia* after an incubation period of 24 h [62]. In this case, the principal phenolic components of lavender oil (linalool, linalyl acetate and 1,8-cineole) determined the antibacterial activity, by acting on the phenolic nucleus and enzyme-dependent reactions of the microorganisms. Lavender EO has been also incorporated into sodium alginate nanofibres for the production of dressings suitable to treat skin burns induced by mid-range ultraviolet radiation [50]. The dressings were effective to inhibit the formation of biofilms of *S. aureus* and to promote the production of pro-inflammatory cytokines in vitro (fibroblast cells) and in vivo (mice). The animals treated with the alginate-lavender EO mats rapidly recovered, without traces of skin inflammation (Figure 4a). In fact, after 24 h from the treatment, the levels of pro-inflammatory cytokines (Interleukin-6 and Interleukin-1β) for treated animals were up to 10 times lower than for untreated ones (Figure 4b,c).

### 3.3. Single Chemical Components

A considerable number of studies are based on the use of pure bioactive chemical compounds obtained from plants, such as curcumin [31,63], asiaticoside [34], shikonin [41], cinnamaldehyde [51,64]. Curcumin (Cur), which is extracted from the root of *Curcuma longa* L., is well-known for its medicinal properties including strong anti-inflammatory, anti-oxidant and anti-cancer activity [63,65,66]. Several works have been published on the electrospinning of curcumin-based fibres for wound healing applications. For instance, PCL-poly(ethylene glycol) fibres loaded with 0.5 wt % of Cur have been tested on open wounds. The composite fibres showed efficiency of 99% and 70% in inhibiting the proliferation of *S. aureus* after 12 and 24 h, respectively [67]. The dissolution of polyethylene glycol (PEG) in aqueous environment was considered positive in facilitating the release of the active agent from the nanofibres. In vivo tests revealed that, after 10 days of treatment, the wound closure rate was of 90% for animals treated with Cur-loaded PCL fibres. Better performances were achieved for the Cur-loaded PCL-PEG mats, with closure rates of 80% and 99% after 5 and 10 days, respectively. In addition, the composite fibres inhibited the production of nitric oxide (NO), which is a major pro-inflammatory mediator in pathogenic infections, in mouse macrophages. In another study, the antibacterial activity of Cur loaded in poly(2-hydroxy ethyl methacry-late), poly (2-hydroxyethyl methacrylate) (p(HEMA)), fibres has been tested against multidrug resistant microorganisms: methicillin resistant *S. aureus* (MRSA), Extended spectrum β-lactamse (ESBL) *E. coli* and *K. pneumonia* [68]. Zones of inhibition of 17 mm were observed for MRSA and of 18 mm for ESBL *E. coli* and *K. pneumonia*, demonstrating that this natural compound can be used against antibiotic-resistant bacteria. In order to control the release of curcumin, core-shell PVA-chitosan fibres have been also produced [69]. It was observed that the release profile of the natural ingredient, which was loaded into the core of the fibres, was characterised by an initial burst release (40% of the drug in few hours) followed by a constant slow release (up to 85%) in 10 days. The core-shell Cur/PVA-chitosan fibres were able to inhibit the proliferation of MRSA and *S. epidermidis* after six days of incubation with viability loss of 92% and 82%, respectively.

Another extract of interest in wound care is shikonin, which is the major component of the dried root of *Lithospermum erythrorhizon*—a Chinese herbal medicine—and possesses a wide spectrum of properties, such as anticancer, antioxidant, anti-inflammatory, and antibacterial activity [70]. Han and co-workers reported on the encapsulation of shikonin into electrospun PCL/poly(trimethylene carbonate) (PTMC) fibres [41]. They observed that the drug was rapidly released from the electrospun mats in the first hours and then at a constant rate for 48 h. The shikonin-loaded PCL/PTMC fibres were tested against *E. coli* and *S. aureus*: after 24 h of contact fibres containing 5 wt % of shikonin showed an inhibition ring of 16.9 and 21.3 mm for *E. coli* and *S. aureus*, respectively. The healing properties of shikonin have been also investigated in combination with alkannin, which is another naturally occurring hydroxynaphthoquinone, for the development of topical/transdermal dressings [71]. In this case, cellulose acetate, poly(lactic acid) (PLA), and two different blends of poly(lactic-co-glycolic) were used as polymer matrices.

## 4. Tissue Engineering

As emerged from the above discussion, medicinal plant extracts have been extensively used in wound management for their limited cytotoxicity, as well as antimicrobial, anti-inflammatory, and anti-oxidant activity. Those characteristics are relevant also in other biomedical areas, including tissue engineering [72,73,74]. For instance, medicinal plants with osteogenic properties—such as *Cissus quadrangularis* [72] and Asian Panax Ginseng root [73]—have been proposed for bone regeneration. The combined effect of *Cissus quadrangularis* (CQ) and hydroxyapatite (HA) have been investigated by producing PCL-CQ-HA electrospun scaffolds [72]. Enhanced adhesion and proliferation of human fetal osteoblasts (hFOBs) on the composite scaffolds were observed. Moreover, increased levels of mineralisation and osteocalcin expression were detected, which are fundamental in bone formation. PCL scaffolds with osteo-inductive potential were produced also using Asian Panax Ginseng root extract [73]. The ginseng extract had a positive effect on the hydrophylicity and mechanical properties of the nanofibres, also inducing osteogenic differentiation of rabbit mesenchymal stem cells (MSCs) and significant expression of osteogenic genes. Selvakumar et al. produced antibacterial scaffolds for guiding bone regeneration using segmented polyurethane (SPU) and *Aloe vera* wrapped mesoporous hydroxyapatite (Al-mHA) nanorods [74]. The Al-mHA nanorods were prepared by sonochemistry and phyto-synthesis route using *Aloe vera* extract, whereas the SPU matrix was based on soft segments of PCL, poly(ethylene carbonate) (PEC) and poly(dimethylsiloxane) (PDMS), PCL-PEC-b-PDMS. SPU/Al-mHA fibres exhibited a diameter of 567 nm and were characterised by improved mechanical properties. When compared with pristine SPU mats, the SPU/AL-mHA scaffolds showed an increase of 50% and 60% in Young’s modulus and the elongation at break, respectively. The ornamented scaffolds were tested against human pathogenic bacteria, such as *E. coli*, *S. Typhi*, *V. cholera*, *P. aeruginosa*, *R. rhodochrous*, *P. vulgaris*, *A. hydrophila*, and *B. cereus*, which are responsible for bone infections (osteomyelitis). Zones of inhibition with a diameter of 10–12 mm were detected for all bacteria analysed. Tests on subcutaneous and intraosseous (tibial) implantation in rabbits proved that the scaffolds prevented infections and promoted cartilage formation, endochondral ossification, and mineralisation.

## 5. Food Industry

In food processing and packaging, electrospun fibres are used for the encapsulation of plant extracts with the aim of preserving the integrity and controlling the release of the active ingredients [75,76]. In those sectors, electrospinning offers the advantage of being a cost-effective manufacturing procedure that operates at room temperature and it is compatible with the majority of edible polymers and materials approved for food contact [77]. For example, electrospun nanofibres of zein (corn protein) have been proposed for the encapsulation of β-carotene [78], which is a natural antioxidant used as a dietary supplement and food colorant, and gallic acid [79], which is a phenolic acid with anti-inflammatory, anti-oxidant, and antibacterial properties. The results demonstrated that the photo-oxidation of β-carotene was remarkably delayed inside the nanofibres and that gallic acid maintained 1,10-diphenyl-2-picrylhydrazyl (DPPH) radical scavenging effect.

In this section, the use of electrospun nanofibres as carriers for plant extracts of interest in food industry and as materials for smart food packaging will be discussed.

### 5.1. Nanofibres as Carriers of Plant Extracts

Electrospun nanofibres of zein [80] and PVA [81] have been proposed as carriers and stabilisers of epigallocatechin-gallate (EGCG), a green tea polyphenol with antioxidant and antimicrobial activity but also unstably prone to oxidation-reduction reaction. Sun et al. have demonstrated that the encapsulation of EGCG into PVA electrospun fibres and its complexation with copper preserved and even enhanced the bactericidal activity of EGCG against *Bacillus cereuse* and *Pseudomonas nitroreducens* [81]. The intrinsic antibacterial properties of EGCG combined with the positive charges of the Cu complex were responsible for modification of the bacterial membrane with leakage of intracellular materials and consequent bacterial death.

**Figure 5:**
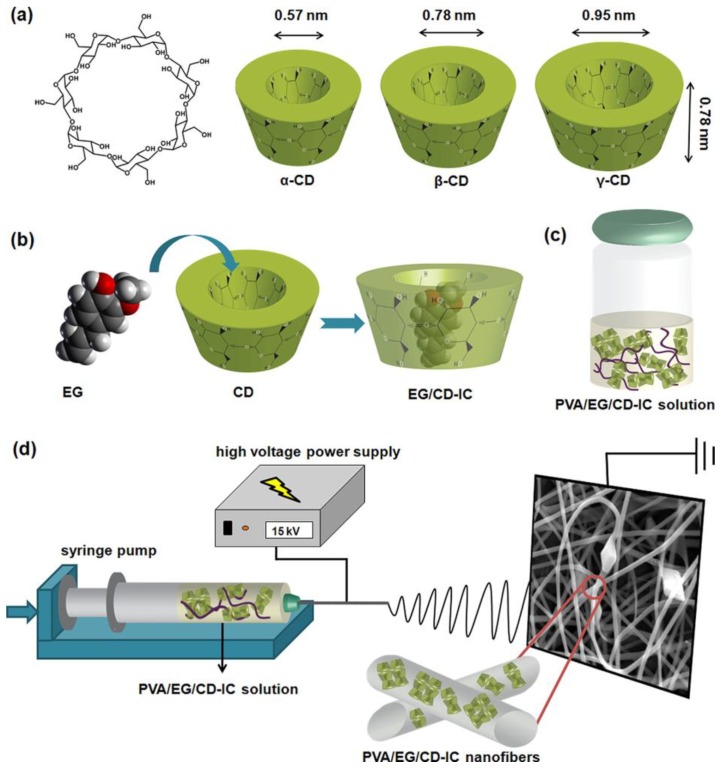
(**a**) Chemical structure of β-CD and dimensions of α-CD, β-CD, γ-CD; (**b**) Schematic representations of (b) the formation of EG/CD-IC; (**c**) the PVA/EG/CD-IC solution; and (**d**) the electrospinning process leading to the production of PVA/EG/CD-IC fibres. Reproduced with permission from [86]. Copyright American Chemical Society, 2013.

In order to prolong the shelf-life, increase the thermal stability, and limit the volatility of natural compounds, cyclodextrin inclusion complexes (CD-ICs) of plant extracts are particularly used in food industry [82,83,84]. CDs are cyclic oligosaccharides with a cavity-shaped structure, composed of six to eight glucopyranose units [75]. They form complexes with hydrophobic compounds and are typically used as drug carrier. Kayaci et al. demonstrated the encapsulation of vanillin/CD-IC into poly(vinyl alcohol) (PVA) mats [85]. Vanillin is a preservative largely used in food industry but highly volatile. The work showed that the weight loss of vanillin from PVA/vanillin/CD-IC fibres was only 42% after 50 days of storage at room temperature, whereas PVA/vanillin exhibited a weight loss of 44% already after 1 day of storage in the same conditions. PVA electrospun meshes encapsulating eugenol (EG) CD-IC were also produced (Figure 5) [86]. EG is a fragrance and a germicide extracted from plants, with limited stability when exposed to oxygen, light, and high temperature. High thermal stability of EG was achieved when EG/CD-ICs were electrospun in a PVA matrix, as demonstrated by the shift of EG degradation temperature at high values and the slow release at elevated temperatures. Similar behaviour was observed also for geraniol CD-ICs loaded into PVA electrospun membranes [87,88]. Geraniol is one of the components of essential oils and characterised by insect repellent, antimicrobial, and antioxidant activity. The study of Aytac et al. reported on the electrospinning of complexes of geraniol with three modified hydroxypropyl and methyl groups CDs (HPβCD, MβCD, and HPγCD) and PVA [88]. They showed that geraniol was well preserved into the nanofibres, with only 24% and 50% (*w*/*w*) release from MβCD/geraniol-IC and MβCD (HPγCD)/geraniol-IC fibres after 50 days of storage at room temperature, respectively. On the contrary, geraniol encapsulated into PVA fibres without CDs was not stable and 71% of the compound evaporated after 50 days. The use of CD complexes also increased the aqueous solubility of the natural extract with positive effects on the antioxidant and antibacterial activity of the CD/geraniol-IC nanofibres: up to 75% of antioxidant activity and 85%–100% of antibacterial activity against two model microorganisms (*E. coli* and *S. aureus*) were achieved. Therefore, the encapsulation of geraniol into CD-IC nanofibres was advantageous in increasing the shelf life of the natural compound, together with its antioxidant and antibacterial properties.

### 5.2. Active Food Packaging

In food packaging applications, functional molecules extracted from plants have been exploited for prolonging food shelf-life and avoiding bacteria colonisation [89,90]. In a recent study, electrospun mats of PVA and β-cyclodextrin (PVA/CEO/β-CD) containing cinnamon essential oil (CEO) have been produced and tested against *S. aureus* and *E. coli* [60]. CEO was chosen because it was more effective against those microorganisms than clove, Artemisia, and eucalyptus EOs. The combination of CEO with β-CD further enhanced the antibacterial action of this essential oil. In fact, the study demonstrated that the fibrous PVA/CEO/β-CD substrates produced large bacteria inhibition zones, probably due to the porosity that facilitated the release of CEO. In disk diffusion tests, PVA/CEO/β-CD electrospun mats showed inhibition zones of 29 and 30 mm for *E. coli* and *S. aureus*, respectively; MIC values of 1.0 and 9.0 mg/mL was measured for *E. coli* and *S. aureus*, respectively. The electrospun membranes were evaluated in strawberry preservation tests and compared with commercial fresh-keeping films (namely, films based on commercial thermoplastics, such as low density polyethylene (LDPE), that can prolong storage life, and maintain better flavour and appearance of food). Unpacked strawberries were easily perishable with a maximum storage life of four days, but when packed with fresh-keeping films the shelf life was extended to six days. On the contrary, the fruit packed with PVA/CEO/β-CD fibres were stable for more than six days without losing their flavour.

Active multilayer packaging systems based on outer layers of compression moulded polyhydroxybutyrate (PHB) films and inner layer of zein electrospun fibres encapsulating cinnamaldehyde (CNMA, the bioactive chemical component of CEO) have been developed [91]. The virucidal activity of CNMA was tested on norovirus surrogates, murine norovirus (MNV) and feline calicivirus (FCV), and hepatitis A virus (HAV). The multilayer films completely inactivated FCV and induced a high reduction of the infectivity of MNV and HAV, demonstrating their potential in preventing food contamination by enteric viruses. In another study, bacterial contaminations of meat foodstuffs have been stopped by hybrid packaging materials composed of electrospun polyurethane nanofibres containing zinc nanoparticles and virgin olive oil [92]. Differently from pristine polyurethane fibres that were not active against *S. aureus* and *Salmonella typhimurium*, fibres with olive oil showed a significant inhibition effect, which was further enhanced by the addition of Zn nanoparticles.

## 6. Conclusions

As discussed in this review, diverse studies have demonstrated that naturally-derived bioactive agents are valid alternatives to synthetic counterparts for applications that include wound management, tissue engineering and food industry (Table 1). In particular, the possibility to encapsulate them into polymeric nanofibres using the electrospinning technique has promoted the manufacturing of scaffolds and membranes with improved antimicrobial activity.

In the field of regenerative medicine, in vitro and in vivo tests have highlighted the efficacy of electrospun scaffolds based on plant extracts in stimulating cell proliferation, controlling inflammation response, and preventing bacteria colonisation. Studies on crude plant extracts, essential oils, or single chemical components all seem to point that their encapsulation in electrospun fibres of carefully chosen polymeric matrices can induce a much-controlled release of the active ingredients. This methodology can ensure that the antimicrobial activity is more effective in the long term, thanks to the slow degradation of the polymer matrix, which progressively expose the active component, and better suited to treat wounds thanks to the biocompatibility of all components. Moreover, the electrospinning of natural ingredients has been proposed for preserving the stability and integrity of food and for developing active packaging systems that prolong food shelf-life and avoid biofilm formation. The use of electrospun fibres containing active ingredients also seems to surpass the commercial fresh-keeping films in efficiency.

It is then foreseeable that future research in the field will aim to combine the results mentioned above with new developments in the field of polymer manufacturing, by creating multifunctional architectures that can exploit the properties of diverse medicinal plants. The use of plant-derived synergistic compounds may also represent an effective approach to tackle health issues related to AMR. In fact, natural extracts can prevent and treat biofilms of multidrug resistant microorganisms, such as MRSA and ESBL *E. coli* and *K. pneumonia*.

## Figures and Tables

**Table 1:** Antibacterial activity of selected plant components contained in electrospun fibres.

Plant Extract	Electrospun Matrix	Microorganisms	MIC (mg/mL)	IZD ^1^ (mm)	Viability Loss (%)	Application	References
*Centella asiatica*	Gelatin	*S. aureus*	6.2	-	-	Wound dressings	[39]
		*E. coli*	25.0	-	-		
		*P. aeruginosa*	25.0	-	-		
Baicalein	Silk fibroin-PVP	*S. aureus*	-	-	88–99	Wound dressings	[37]
Green tea	Chitosan-PEO	*S. aureus*	-	6.0	-	Wound dressings	[36]
		*E. coli*	-	4.0	-		
*Garcinia mangostana*	CS-EDTA/PVA	*S. aureus*	0.5–2.0	-	-	Wound dressings	[42]
		*E. coli*	0.5–2.0	-	-		
*Tecomella undulata*	PCL-PVP	*P. aeruginosa*	-	30.0	-	Wound dressings	[32]
		*S. aureus*	-	24.0	-		
		*E. coli*	-	28.0	-		
Chamomile	PCL-PS	*S. aureus*	-	7.6	-	Wound dressings	[35]
		*C. albicans*	-	7.6	-		
Cinnamon EO	Chitosan-PEO	*E. coli*	-	-	80–99	Wound dressings	[51]
		*P. aeruginosa*	-	-	48–81		
Cinnamon EO	PVA	*E. coli*	1.0	28.9	-	Food packaging	[61]
		*S. aureus*	0.9	30.5	-		
Cinnamon EO Peppermint EO Lemon grass EO	Cellulose acetate	*E. coli*	-	-	-	Wound dressings	[46]
*Syzygium aromaticum* oil	PAN	*S. aureus*	-	25.0–28.0	-	Wound dressings Tissue engineering	[61]
		*B. subtilis*	-	25.0–28.0	-		
		*K. pneumonia*	-	18.0–20.0	-		
		*E. coli*	-	18.0–20.0	-		
Lavender EO	PAN	*S. aureus*	0.1	14.0–15.0	-	Wound dressings Tissue engineering	[62]
		*K. pneumonia*	0.1	14.0–15.0	-		
Lavender EO	Alginate	*S. aureus*	-	20.0	-	Wound dressings	[50]
Curcumin	PCL-PEG	*S. aureus*	-	-	99	Wound dressings	[67]
Curcumin	p(HEMA)	MRSA	-	17.0	-	Tissue engineering	[68]
		ESBL *E. coli*	-	18.0	-		
		ESBL *K. pneumonia*	-	18.0	-		
Curcumin	PVA-Chitosan	MRSA	-	-	92	Tissue engineering	[69]
		*S. epidermidis*	-	-	82		
Shikonin	PCL/PTMC	*S. aureus*	-	21.3	-	Wound healing	[41]
		*E. coli*	-	16.9	-		
*Aloe vera*	Polyurethane	*E. coli*, *S. Typhi*, *V. cholera*, *P. aeruginosa*, *R. rhodochrous*, *P. vulgaris*, *A. hydrophila* and *B. cereus*	-	10.0–12.0	-	Bone regeneration	[74]
*EGCG-Cu^II^*	PVA	*B. cereuse*	8.0	-	-	Food industry	[81]
		*P. nitroreducens*	20.0	-	-		
*Geraniol*	PVA	*S. aureus*	-	-	85-100	Food industry, cosmetics	[88]
		*E. coli*	-	-			

^1^ IZD: Inhibition zone diameter.
